# Delayed Anterior Cervical Screw Migration Causing Swallowing Difficulty

**DOI:** 10.7759/cureus.16208

**Published:** 2021-07-06

**Authors:** Timothy Maurer, Robert Maurer, George T Reiter

**Affiliations:** 1 Osteopathy, Philadelphia College of Osteopathic Medicine, Philadelphia, USA; 2 Neurosurgery, Penn State University College of Medicine, Milton S. Hershey Medical Center, Hershey, USA

**Keywords:** cervical, spine, hardware failure, dysphagia, acdf

## Abstract

Cervical disc disease is a common pathology that may require surgical intervention. A common surgical intervention for cervical disc disease is anterior discectomy and fusion. A number of complications are associated with this technique, including hoarseness, dysphagia, pseudoarthrosis, adjacent segment disease, and hardware failure. Here, we present a case detailing a unique complication of delayed migration of an anterior cervical screw which resulted in swallowing difficulties. Subsequently, we discuss the related reports and highlight the literature on the topic. We conclude with a brief discussion on the principle of patient autonomy.

## Introduction

Cervical disc disease affects an estimated 84 per 100,000 individuals in the United States [1]. This commonly presents as stenosis manifesting clinically as radicular pain or myelopathy that can require surgical intervention if activities of daily living are impaired. For many patients with cervical disc disease, treatment involves an anterior cervical discectomy with fusion (ACDF). This procedure, first outlined in 1955, involves fixation of adjacent cervical vertebrae with a rigid plate and screws [2]. More recently, a fusion construct has been developed in which the plate sits in the anterior aspect of the disc space with screws oriented obliquely to penetrate the anterior portion of the endplates. This case study describes a unique postoperative complication of this surgery and its progression as documented by serial imaging.

## Case presentation

A 69-year-old male presented to the clinic with a two-year history of slowly progressive gait dysfunction manifesting as increasing clumsiness in his lower extremities which affected his ability to play recreational sports and resulted in several falls. In addition, the patient reported a two-month history of increasing neck pain but denied upper extremity radiculopathy. The patient also denied any inciting trauma. Notable findings on the initial physical examination revealed a bilateral Babinski sign and clonus at both ankles. Motor and sensory functions were intact throughout. MRI revealed severe central cervical stenosis of C3-4 and C4-5 with associated retrolisthesis. Preoperative upright flexion-extension X-ray did not show any instability. The patient declined surgical intervention against medical advice but agreed to continued observation. At the six-month and two-year follow-ups, the patient reported similar symptoms without any significant progression. At a subsequent follow-up, the patient reported that he had undergone a C3-4 and C4-5 ACDF procedure at an outside institution. While he did not note any complications, he also did not note any significant improvement in his symptoms.

**Figure 1:**
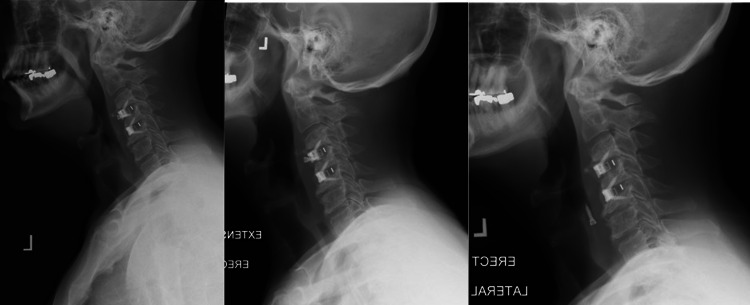
Cervical radiographic images. (A) Postoperative X-ray one year following C3-4, C4-5 ACDF. (B) Postoperative X-ray 17 months following ACDF. (C) Postoperative X-ray 18 months following ACDF. Note the caudal screw at C3-4 fusion progressively backs out until the screw is observed free-floating in the soft tissue. ACDF: anterior cervical discectomy with fusion

One year after the surgery, the patient returned to our institution with continued complaints of gait instability and balance issues. X-ray imaging at this time revealed that the caudal fixation screw of the C4 fusion was not fully seated in the plate (Figure 1A). Five months later, the patient presented with complaints of difficulty swallowing in addition to balance issues. A repeat X-ray image revealed that the previously noted caudal fixation screw at C4 had backed further out of the plate (Figure 1B). The patient again declined surgical intervention and continued follow-up was arranged. One month later, the patient returned for follow-up. He reported no change in his symptoms with persistent swallowing difficulty. His lateral cervical X-ray at this time revealed that the C4 screw had fully migrated out of the plate and was resting in the soft tissue anterior to the vertebral body (Figure 1C). ENT was consulted for possible removal of the screw but the patient declined surgical intervention in favor of continued clinical follow-up.

## Discussion

ACDF is a widely utilized procedure that historically has been associated with a low rate of morbidity. Nonetheless, given the complexity of anatomy in the region, complications are broad and can be fatal if not rapidly recognized. Some of the more common early and late postoperative complications include adjacent segment disease (8.1%), dysphagia (5.3%), C5 nerve root palsy (3%), graft or hardware failure (2.1%), pseudarthrosis (2.0%), and recurrent laryngeal nerve palsy (1.3%) [3]. This case report details the presentation of a rare form of hardware failure in which one of the fixation screws backed out of the plate entirely, endangering nearby soft tissue structures and manifesting clinically as dysphagia. The locking mechanism designed to hold the screw in the plate failed which allowed the screw to completely back out and become lodged in the surrounding soft tissue. This failure could be a result of a defective locking mechanism within the plate or the failure of the surgeon to trigger this locking mechanism. The combination of the locking mechanism failure and a nonunion most likely caused the screw to back out.

In this case, the patient returned to our institution following a C3-4 and C4-5 ACDF at another hospital because some of his preoperative symptoms did not improve. His radiologic findings at the time revealed that the caudal screw at the C3-4 interspace was not fully countersunk; therefore, it could be postulated that it was not properly locked into the plate and at risk of backing out. At the time, he did not have swallowing issues. Observational follow-up five months later, when the patient developed swallowing difficulties, revealed that the screw had backed out further, confirming that the locking mechanism had failed. In the subsequent month, the screw continued to back out until it was free from the bone and plate and was sequestered in the adjacent soft tissue.

Early hoarseness is common after ACDF and increases in frequency when multiple levels are fused, most likely due to increased dissection, tissue stretch, and longer operative times [4]. Fortunately, the major complication of a pharyngoesophageal injury is much rarer, with an estimated incidence of approximately 0.2% [5-16]. As in this case, complaints of dysphagia that develop several months after ACDF surgery should be evaluated with expeditious radiological evaluation, as these complaints may indicate a late pharyngoesophageal injury. For instance, in one report, a dislocation of bone graft used in the fusion eroded through the esophageal wall and became lodged in the pharynx [12]. In another case, a 76-year-old woman was discovered to have coughed up a screw that had backed out following an ACDF procedure [13]. Complications related to hardware may lead to erosions in the esophagus, trachea, or major vasculature resulting in the formation of fistulas, infections, or hemorrhages. Such complications may pose a 20% mortality rate if recognized and treated within the first 24 hours but up to 50% if treatment is delayed [4,10,17]. Therefore, early and late pharyngoesophageal injuries are among the most serious complications of ACDF procedures, despite their relative rarity.

The current case is unique in the literature for its dramatic findings on imaging which document the progression of the screw migration out of the plate, beginning a year postoperatively and progressing over the next six months. While asymptomatic hardware failure can be reasonably monitored, when symptomatic, surgical correction is usually indicated. For the case discussed here, that would have entailed a removal and/or revision of the displaced screw when the patient developed swallowing difficulties. However, the patient’s decision to forego surgery in favor of clinical follow-up permits an unusual clinical scenario where the mechanical displacement of the screw can be observed over time.

While the current case demonstrates dramatic imaging studies chronicling the progression of the screw displacement, a number of other reports have highlighted the possibility of developing delayed hardware complications. A recent report from Quadri et al. described an 81-year-old female who expectorated an entire anterior cervical fusion construct three and half years after her surgery [18]. Similarly, a 76-year-old female was reported as expectorating a screw more than five years after her anterior cervical fusion [13]. Among the most unusual reports of delayed hardware complication was by Fujibayashi et al. who described the case of a patient whose anterior cervical fusion construct seemingly disappeared without a trace and was figured to have eroded into the gastrointestinal tract and passed into the feces without morbidity [19]. Although dramatic in nature, such reports highlight the possibility of delayed hardware failure and the importance of clinician awareness.

The case also highlights the important concept of patient autonomy. As providers, it is crucial to counsel patients in clear and comprehensible language regarding their medical options [20]. However, in patients who possess insight, understanding, and capability to make their own medical decision, the final choice ultimately belongs to the patient. In such cases, it is the provider’s responsibility to coordinate continued care in line with the patient’s wishes as further developments may occur in their clinical course which would change management.

## Conclusions

In conclusion, hardware failure, dysphagia, hoarseness, and pharyngoesophageal injury are all possible complications of ACDF procedures. These may present in an immediate fashion or may be delayed. Even when swallowing difficulties develop months or even a year after ACDF, a thorough workup, including imaging studies, should be initiated to ensure there are no slowly developing complications associated with the hardware.
